# Treatment of a giant terminal internal carotid artery aneurysm in a 10-year-old child by flow diversion: long-term outcome and lessons learned

**DOI:** 10.1093/bjrcr/uaaf024

**Published:** 2025-03-28

**Authors:** Wilhelm Kuker, Jayaratnam Jayamohan

**Affiliations:** Department of Clinical Neuroscience, University of Oxford, Oxford OX3 9DU, United Kingdom; Department of Clinical Neuroscience, University of Oxford, Oxford OX3 9DU, United Kingdom

**Keywords:** aneurysm, stents, child, hyperplasia complications

## Abstract

A 28-mm diameter and partially thrombosed intracranial aneurysm was found in a 10-year-old boy on an MRI for non-specific headaches. The large neck incorporated the left internal carotid artery (ICA) termination and proximal middle cerebral artery. Treatment was planned to prevent further growth and rupture. Because of the difficult anatomy, a braided stent was first placed across the aneurysm neck as a scaffold to allow for the placement of a flow-diverting stent after its endothelialisation. However, severe stent-induced endothelial hyperplasia was encountered when the flow diverter was inserted. This resulted in a transient ICA occlusion during the procedure before flow was restored by angioplasty. As a result, the patient suffered a mild transient dysphasia but permanent loss of vision in the left eye. All antiplatelet medications were stopped 1 year after the procedure without problem. The aneurysm has remained fully occluded in the 7 years since.

## Introduction

Intracranial arterial aneurysms are a common cause of spontaneous subarachnoid haemorrhage in adults and unruptured aneurysms have an incidence of around 3%.^1^ In contrast, they are rare in children. A review found only 573 paediatric patients, mostly boys, with intracranial aneurysms published over 15 years.^2^^,^^3^ Compared to adults, there is a larger proportion of giant aneurysms in children, presenting with mass effect^4^ instead of acute rupture. Aneurysms in children also seem pathophysiologically^5^ different and intracranial dissections have been implicated as a frequent cause.

The treatment of large, giant and fusiform intracranial aneurysms is based on the exclusion of the aneurysm from the blood circulation.^6^ The easiest approach is the endovascular occlusion of the parent artery which can often be achieved with a very low risk of complications. This is called the deconstructive approach. However, it requires an adequate collateralisation of the occluded parent vessel. This treatment can often be used for large aneurysms of the internal carotid or the vertebral arteries.^7^

If the aneurysm originates from a brain artery beyond the circle of Willis, the deconstructive approach will lead to cerebral infarctions.

Then the aneurysm must be excluded from circulation while preserving the flow in the parent artery (reconstructive approach).

This can be achieved by placing a flow-diverting (FD) stent. This is a woven metal stent with high wall coverage, which slows blood flow in the aneurysm and induces thrombosis there while maintaining blood flow in the parent artery. Flow diversion has been shown to achieve good aneurysm occlusion rates in adults with an acceptable complication^8^ risk.

In contrast, data to guide the treatment of giant aneurysms in children are very limited.

Therefore, we would like to share the treatment of an unruptured giant aneurysm in a 10-year-old boy with a FD stent. The aneurysm was successfully excluded from circulation and has remained occluded at 7 years follow-up. However, the treatment was complicated by massive intimal hyperplasia, uncommon in adults. Operators should be aware of this problem and take measures to avoid complications.

## Case report

**Figure 1.**
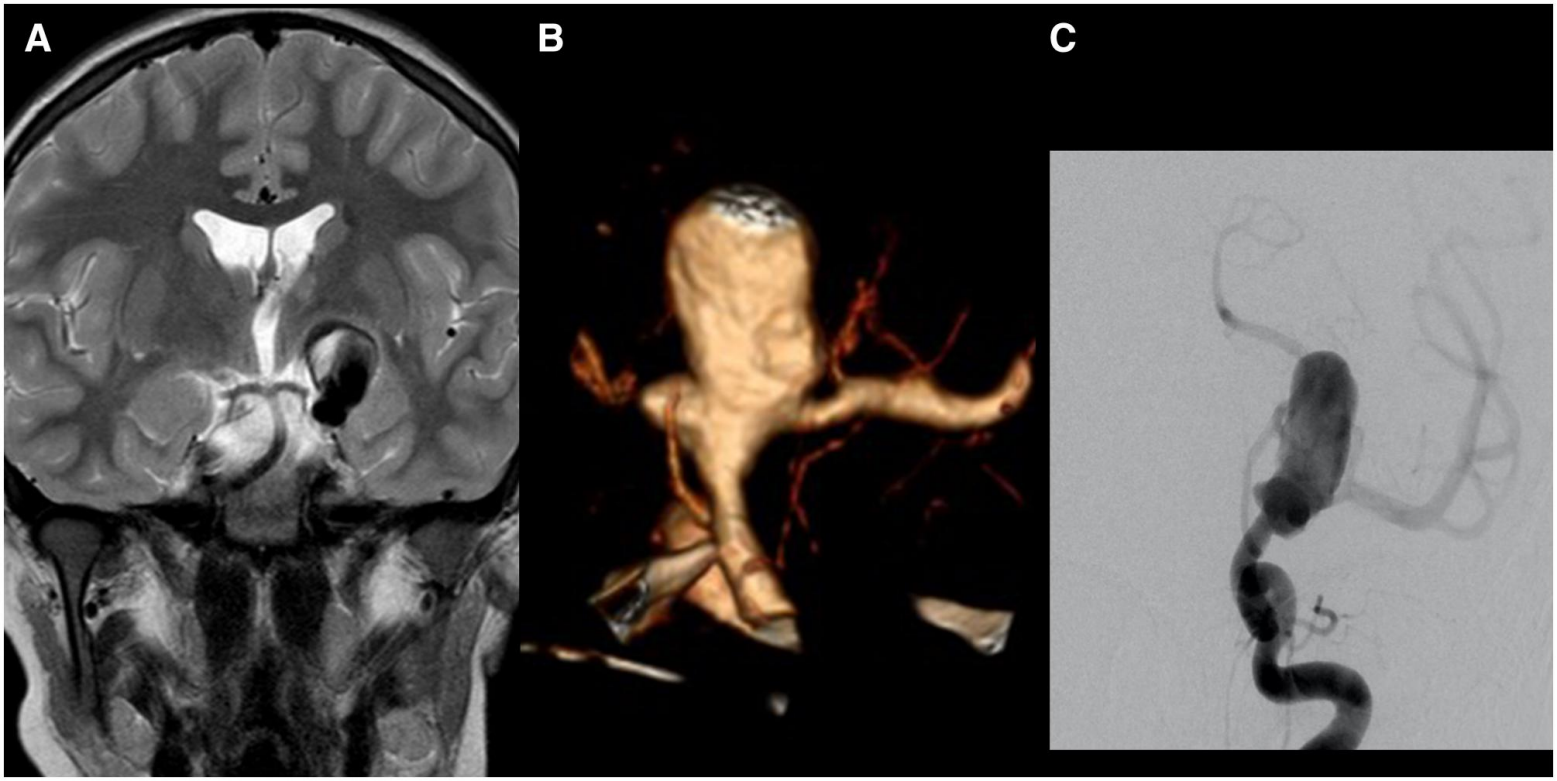
(A) Coronal T2-weighted image of an MRI performed for unspecific headache in the then 10-year-old boy. It shows a giant and partially thrombosed aneurysm of the left ICA. There is no oedema but mass effect on the third ventricle. (B) The CTA shows the broad-based aneurysm incorporating the carotid termination. There is no A1 segment. The left M1 segment originates from the aneurysm. A smaller lobule is seen pointing medially. The internal surface of the aneurysm appears coarse. (C) The diagnostic angiogram shows contrast pooling in the aneurysm before filling theMCA. Abbreviation: ICA = internal carotid artery; CTA = CT angiography; MCA = middle cerebral artery.

A 10-year-old previously healthy boy underwent an MRI scan for non-specific headaches. This showed a 28 mm partially thrombosed aneurysm incorporating the left internal carotid artery (ICA) termination and the proximal M1 segment of the middle cerebral artery (MCA) into its neck (Figure 1A). There was considerable associated mass effect. Concomitant signs of established basal ganglia infarction suggested an underlying chronic dissection, not diagnosed at the time of its occurrence.

A CT angiogram was performed for further delineation of the neck and for procedure planning. This study confirmed the presence of a very broad aneurysm neck and the absence of the left A1 segment (Figure 1B). The small posterior communicating artery is connected to the left ICA below the aneurysm.

After a discussion with the regional paediatric neurosurgical centre, it was decided for the boy to be treated by a team familiar with the management of complex intracranial aneurysms in adults. It was felt necessary to secure the aneurysm to prevent further growth, symptomatic mass effect and rupture.

It was determined that a deconstructive approach with parent artery occlusion would require bypass surgery because the aneurysm neck incorporated the proximal M1 segment, extending beyond the circle of Willis. In addition, the posterior communicating artery appeared very small on CT angiography (CTA) and the left A1 segment was absent.

A reconstructive approach with coil embolization and flow diversion was felt to be safer to permanently occlude the aneurysm while preserving the patency of the MCA.

Avoiding the migration of an FD stent into a large aneurysm requires the stable distal anchorage of the device. Here, this would have required to placing the distal end of the FD deep into the M1, thereby covering the lenticulostriate perforators. This would have risked their occlusion and more damage to the basal ganglia.

While a non-FD stent would also need to extend past the origin of the lenticulostriate vessels for secure distal fixation, it would preserve their patency.

The plan was therefore to initially bridge the aneurysm neck with a braided stent and coil the aneurysm. Later the flow diverter would be placed into this scaffold to cover the aneurysm neck, but not the perforating branches.

The placement of the flow diverter would be scheduled after stent endothelialization.

This staged endovascular treatment approach was accepted by the patient’s parents.

The patient was started on 75 mg of aspirin and clopidogrel each per day, commencing 5 days before the procedure.

## First procedure

Under general anaesthesia, the right femoral artery was punctured and a 5F catheter sheath was inserted. A 5F Envoy catheter was placed into the left ICA. The diagnostic angiogram confirmed the CTA findings (Figure 1C). The absence of the A1 segment and the location of the aneurysm distal to the small posterior communicating artery precluded parent artery occlusion.

Placing a Vasco 21 microcatheter into the MCA beyond the aneurysms required an exchange procedure as it was only possible to cannulate the M1 segment with an Echelon 10 Synchro 14 combination.

There was high friction in the aneurysm neck suggesting a rough vessel wall.

**Figure 2.**
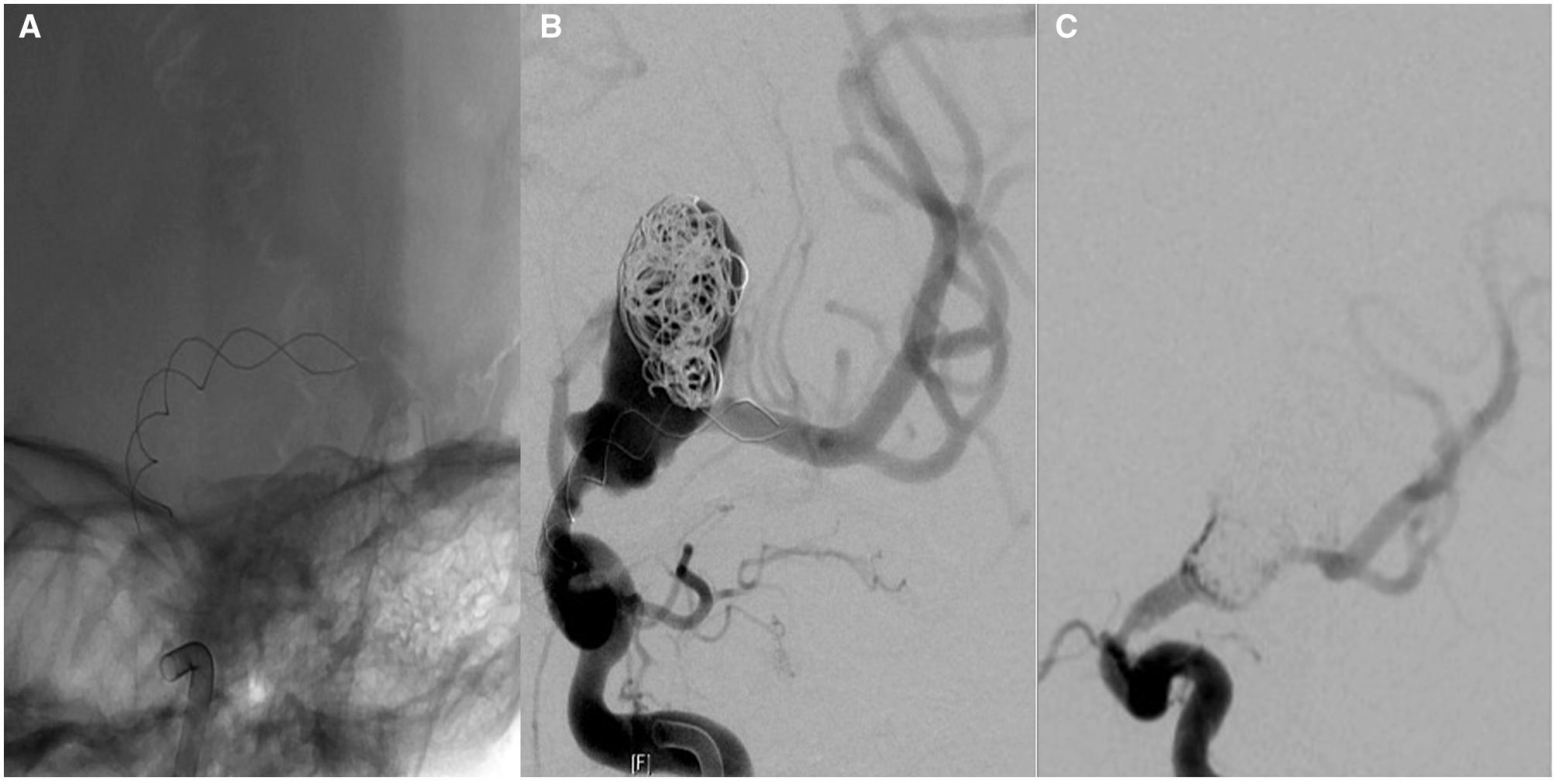
(A) A Leo 3.5 × 35 mm braided stent was deployed across the aneurysm neck with some difficulty. The cannulation of the M1 was impaired by the rough lining of the aneurysm and the acute angle of the M1 exit. (B) Some coils were added during the same sitting. Placement of a flow-diverting stent would have required the passage of the stent which was regarded as too risky due to possible stent dislocation into the aneurysm. (C) Four months later, the aneurysm was packed more densely with coils. No stent was added because of intimal hyperplasia in the Leo stent.

After placement of a Leo 3.5 × 35 mm stent from the left M1 into the supra ophthalmic ICA (Figure 2A), some coils were inserted into to aneurysm (Figure 2B). Loose coil packing was accepted to avoid displacing the stent after the difficult deployment.

The patient recovered from the procedure without neurological deficit.

The second treatment procedure was scheduled 4 months later. The plan was to place a SILK FD stent into the now fully endothelialized and stable Leo stent.

## Second procedure

The procedure was again performed under general anaesthesia (GA) with femoral access and through an Envoy guide catheter. The diagnostic angiogram at the beginning of the procedure showed coil compaction but also unexpected intimal hyperplasia at the proximal end of the Leo stent causing a severe ICA stenosis (Figure 2C). The deployment of the flow diverter was therefore deemed too risky, but coils were added into the recurrent aneurysm without problems.

The rest of the procedure was uneventful. The patient awoke without new neurological deficit.

Six months later (10 months after the first treatment), an MRI scan showed further aneurysm recanalization and there was a strong desire to permanently occlude the aneurysm. Hence another attempt was undertaken to deploy an FD stent.

## Third procedure

**Figure 3.**
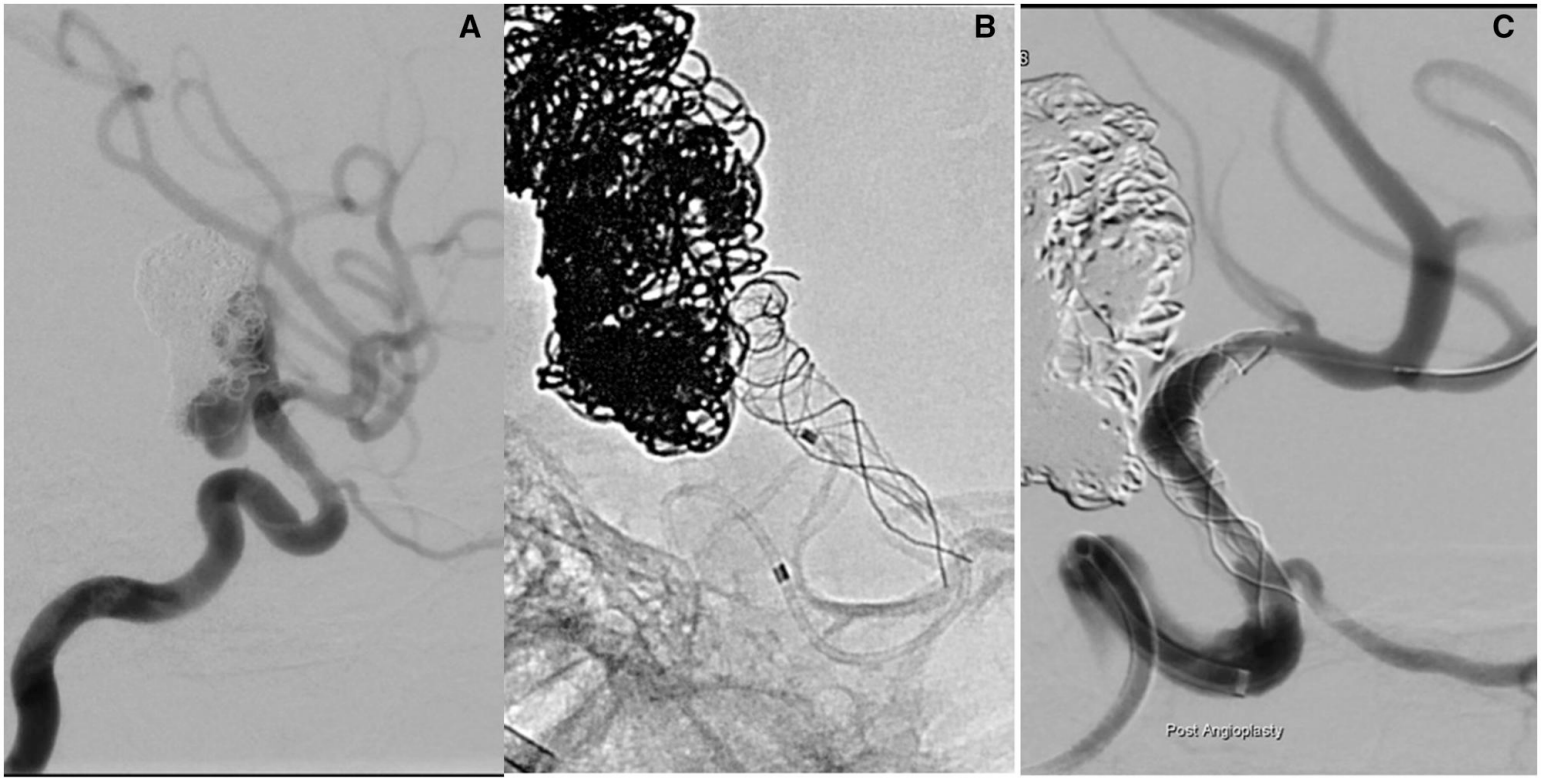
(A) An additional 6 months later, the boy returned for further treatment as an MRI had shown aneurysm recurrence. The diagnostic angiogram was thought to show resolution of the intimal changes. It was possible to advance a microcatheter into the left M1 without problems. (B) Deployment of the SILK flow-diverting stent. The proximal end of the stent does not open. The ICA occludes and it is not possible to advance the microcatheter over the stent delivery wire. (C) After angioplasty with a Maverick coronary balloon flow is restored after 5 h. There remains a significant residual stenosis. Abbreviation: ICA = internal carotid artery.

Under GA and after right femoral puncture an Envoy catheter was placed into the left ICA. The angiogram confirmed the aneurysm recurrence. Compared to the procedure 4 months earlier, the intimal hyperplasia had mostly resolved (Figure 3A). Now it was no longer regarded as a contraindication to placing the FD stent.

An M2 branch was cannulated with a Vasco 21 microcatheter through the pre-existing Leo stent. Then a 3.5 × 20 mm SILK FD stent was released from the M1 segment by retracting the microcatheter. It was placed into the previously deployed braided stent across the aneurysm neck and ended in the ICA below the aneurysm.

However, upon deployment, it became clear that there was considerable and previously unappreciated intimal hyperplasia within the ICA. Thus, the constrained expansion of the SILK resulted in unintended stent lengthening. The proximal end of the SILK then extended into the most severely stenosed segment of the ICA and failed to open after deployment (Figure 3B). This resulted in an occlusion of the left ICA.

Due to the shape of the proximal end of the SILK stent (fish mouthing), it was impossible to re-enter the collapsed stent with the microcatheter to deploy an exchange wire. This would have allowed to advance a balloon for angioplasty. Hence, the SILK deployment wire had to be removed. Further attempts to canulate the proximal end of the SILK with a Synchro 14 wire to place an Eclipse 2L balloon were futile as the balloon could not be pushed past the collapsed proximal end of the stent due to high friction.

Only after the replacement of the Envoy guide catheter with a distal access catheter was it possible to gain access to the lumen of the SILK stent with a Synchro 14 guide wire. This allowed to advance a Maverick balloon into the FD. This coronary angioplasty balloon has a more acute tip and better push ability than an aneurysm remodelling balloon.

The balloon inflation restored blood flow in the left ICA and MCA after 5 h but a severe residual stenosis remained (Figure 3C).

A subsequent CT did not show an infarct.

After the CT and back in the angio room the ICA and MCA were still patent with good flow and no further treatment was performed because of the perceived risk of a reperfusion injury.

The boy was subsequently extubated with some new, mild transient dysphasia but without a motor deficit.

However, he was found to have a loss of central vision in his left eye due to a retinal artery occlusion.

A subsequent brain MRI showed an additional small left basal ganglia infarct. The aneurysm was excluded from circulation after the placement of the FD.

**Figure 4.**
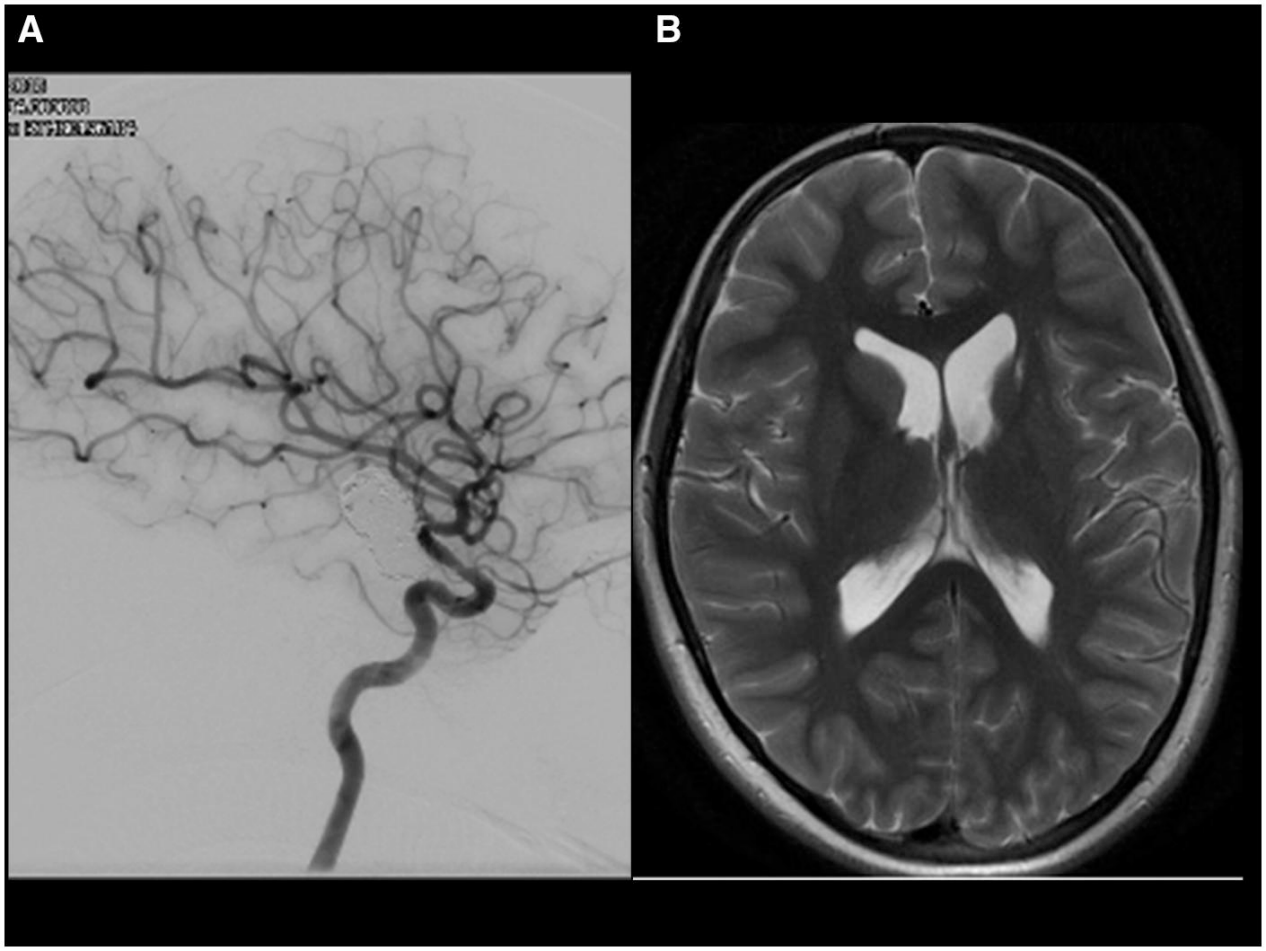
(A) The check angiogram 18 months after insertion of the flow-diverting stent shows aneurysm occlusion with a persistent in stent stenosis. (B) The MRI 12 months later and after discontinuation of all antiplatelet agents shows post ischaemic atrophy of the left-sided basal ganglia. The aneurysm has further shrunk. There was no new ischaemia (not shown).

A cerebral angiogram 18 months after the placement of the SILK stent (Figure 4A) confirmed the occlusion of the aneurysm and showed a mild residual left ICA stenosis due to persistent intimal hyperplasia. There was good and timely perfusion of the left MCA territory. The lenticulostriate arteries originate distal to the FD stent.

All antiplatelet medication was discontinued 1 year after the placement of the FD SILK stent to facilitate aneurysm occlusion. There were no ischaemic events relating to the discontinuation of the antiplatelet medication.

The recent MRI scan 7 years after flow diversion has confirmed persistent aneurysm occlusion (Figure 4B).

## Discussion

Although the relative incidence of giant aneurysms is higher in children than in adults, the overall incidence is very low owing to the overall small number of intracranial aneurysms in this age group. Hence, there is a lack of evidence to determine the best treatment strategy in these patients.

While the patient presented here did not suffer a bleed, his headaches and the size of the aneurysm were regarded as indication for treatment. Endovascular treatment was performed according to an adult paradigm. It was not thought to be possible to occlude this giant aneurysm with coils alone and flow diversion seemed a better approach.

Although at the time there were few reports regarding the use of FD stents in children, we felt justified to use this technique to attempt lasting aneurysm occlusion^9^ and avoid repeated coiling procedures.

To safely place an FD stent while avoiding stent migration into the aneurysm and occlusion of lenticulostriate perforating vessels we initially placed a scaffolding stent across the aneurysm neck. This would allow the precise insertion of a much shorter FD stent, only covering the aneurysm neck, and avoiding the perforators.

Endothelialization of the first stent was expected to allow safe cannulation and deployment of the flow diverter. However, the massive intimal proliferation observed 4 months after stenting would have been highly unusual in an adult.

Even after interval improvement, there was significant residual stenosis which was not recognised during an angiogram.

This caused severe problems during the deployment of the FD stent resulting in the transient ICA occlusion.

This could have been avoided with the acquisition of a 3D angiogram or an angioplasty before the deployment of the FD stent.

The relative lack of attributable permanent left hemispheric deficits may be due to the patient’s age and good leptomeningeal collaterals.

After the procedure, an ophthalmological investigation revealed new loss of central vision in the left eye due to the occlusion of the retinal artery from which he has only partially recovered. The origin of the ophthalmic artery is covered by the Leo stent, but not by the flow diverter.

No predisposing condition for intimal hyperplasia was discovered despite thorough investigation. A recent neurological follow-up examination showed no additional focal deficits. However, the patient still experiences increased fatigue and some word finding difficulties when tired.

Aspirin medication was discontinued after 6 months and in addition clopidogrel a year after the insertion of the FD stent.

Seven years on, the aneurysm remains excluded from circulation, the stent construct patent, and the patient free of new ischaemic symptoms.

## Learning points

- Occlusion of large or giant intracranial aneurysms in children is feasible with flow-diverting (FD) stents.
- The spectrum of possible complications may differ from adults and more data are required to guide treatment decisions.
- Care should be taken when deploying an FD stent in the presence of intimal hyperplasia. The true lumen of the artery may be overestimated on conventional digital subtraction angiography (DSA) images and a 3D acquisition should be considered for better assessment.

## References

[1] Vlak MH , Algra A, Brandenburg R, Rinkel GJ. Prevalence of unruptured intracranial aneurysms, with emphasis on sex, age, comorbidity, country, and time period: a systematic review and meta-analysis. Lancet Neurol. 2011;10:626-636. 10.1016/s1474-4422(11)70109-0 21641282

[2] Beez T , Steiger H-J, Hänggi D. Evolution of management of intracranial aneurysms in children: a systematic review of the modern literature. J Child Neurol. 2016;31:773-783. 10.1177/0883073815609153 26516106

[3] Krings T , Geibprasert S, TerBrugge KG. Pathomechanisms and treatment of pediatric aneurysms. Childs Nerv Syst. 2010;26:1309-1318. 10.1007/s00381-009-1054-9 20033187

[4] Bhogal P , Pérez MA, Wendl C, Bäzner H, Ganslandt O, Henkes H. Paediatric aneurysms—review of endovascular treatment strategies. J Clin Neurosci. 2017;45:54-59. 10.1016/j.jocn.2017.08.009 28887074

[5] Sorteberg A , Dahlberg D. Intracranial non-traumatic aneurysms in children and adolescents. Curr Pediatr Rev. 2013;9:343-352. 10.2174/221155281120100005 24696670 PMC3970571

[6] Trevisi G , Benato A, Ciaffi G, Sturiale CL. Treatment strategies and outcomes for intracranial fusiform aneurysms: a systematic review and meta-analysis. Neurosurg Rev. 2024;47:866. 10.1007/s10143-024-03118-0 39570441

[7] Boisseau W , Darsaut TE, Fahed R, et al Endovascular parent vessel occlusion versus flow diversion in the treatment of large and giant aneurysms: a randomized comparison. World Neurosurg. 2024;185:e700-e712. 10.1016/j.wneu.2024.02.114 38417622

[8] Becske T , Kallmes DF, Saatci I, et al Pipeline for uncoilable or failed aneurysms: results from a multicenter clinical trial. Radiology. 2013;267:858-868. 10.1148/radiol.13120099 23418004

[9] Pierot L , Cognard C. Does stent-assisted coiling still have a place in the management of intracranial aneurysms? AJNR Am J Neuroradiol. 2013;34:1993-1995. 10.3174/ajnr.a3614 23744691 PMC7965421

